# Transdiagnostic Constellations in 2p15–p16.1 Microdeletion Syndromes

**DOI:** 10.1192/j.eurpsy.2026.11261

**Published:** 2026-07-31

**Authors:** A. A. Rehman, N. T. Choudhury, B. R. Carr

**Affiliations:** University of Florida, Gainesville, FL, United States

## Abstract

**Introduction:**

The 2p15–p16.1 microdeletion syndrome presents an instructive window into the convergence of multiple neurodevelopmental and neuropsychiatric traits spanning autism spectrum features, obsessive-compulsive tendencies, tic-like stereotypies, emotional dysregulation, and cognitive inflexibility. Reported cases often exhibit phenotypic constellations that blur conventional nosological boundaries, underscoring the need for transdiagnostic conceptual models.

**Objectives:**

To propose and support a phenotypic constellation model for 2p15–p16.1 microdeletion syndrome grounded in trait interaction rather than additive comorbidity as a means of improving neuropsychological conceptualization and clinical assessment.

**Methods:**

We conducted a synthesis of genomic, neuropsychological, and neuroimaging literature (PubMed, PMC), identifying key phenotypic features across published cases. Review included analysis of structural brain abnormalities (e.g., cerebellum, pons, corpus callosum) and genetic loci within the deleted region (notably BCL11A, USP34, XPO1) implicated in neural development and affective-cognitive regulation.

**Results:**

A consistent pattern of transdiagnostic features emerged:

- Language delays and cognitive inflexibility with perseverative or compulsive behaviors.
- Emotional dysregulation which was not easily captured by single diagnostic frameworks.
- Neuroimaging findings of cerebellar and pontine hypoplasia, callosal anomalies, and microcephaly—frequently linked to BCL11A haploinsufficiency.
- Genetic disruption of networks involved in both affective regulation and neurodevelopment.
- High phenotypic variability, even within small deletions, suggesting complex gene-gene interaction over single-gene causality.

**Conclusions:**

The 2p15–p16.1 microdeletion syndrome exemplifies the need for a transdiagnostic, interactional framework in neuropsychological assessment. Recognizing constellations of traits, rather than fitting individuals into rigid diagnostic categories, offers a more accurate and clinically useful understanding. This approach informs treatment planning in complex cases and illustrates how genomic disruptions can reshape our understanding of psychiatric syndromes as emergent, interactional patterns rather than discrete entities.

**Disclosure of Interest:**

None Declared

